# Trypanocidal Effect of Isotretinoin through the Inhibition of Polyamine and Amino Acid Transporters in *Trypanosoma cruzi*

**DOI:** 10.1371/journal.pntd.0005472

**Published:** 2017-03-17

**Authors:** Chantal Reigada, Edward A. Valera-Vera, Melisa Sayé, Andrea E. Errasti, Carla C. Avila, Mariana R. Miranda, Claudio A. Pereira

**Affiliations:** 1 Laboratorio de Parasitología Molecular, Instituto de Investigaciones Médicas “A. Lanari”, IDIM-CONICET, Universidad de Buenos Aires, Buenos Aires, Argentina; 2 Instituto de Farmacología, Facultad de Medicina, Universidad de Buenos Aires, Ciudad Autónoma de Buenos Aires, Argentina; 3 Department of Pharmacy, University of São Paulo, São Paulo, SP, Brazil; Liverpool School of Tropical Medicine, UNITED KINGDOM

## Abstract

Polyamines are essential compounds to all living organisms and in the specific case of *Trypanosoma cruzi*, the causative agent of Chagas disease, they are exclusively obtained through transport processes since this parasite is auxotrophic for polyamines. Previous works reported that retinol acetate inhibits *Leishmania* growth and decreases its intracellular polyamine concentration. The present work describes a combined strategy of drug repositioning by virtual screening followed by *in vitro* assays to find drugs able to inhibit *Tc*PAT12, the only polyamine transporter described in *T*. *cru*zi. After a screening of 3000 FDA-approved drugs, 7 retinoids with medical use were retrieved and used for molecular docking assays with *Tc*PAT12. From the docked molecules, isotretinoin, a well-known drug used for acne treatment, showed the best interaction score with *Tc*PAT12 and was selected for further *in vitro* studies. Isotretinoin inhibited the polyamine transport, as well as other amino acid transporters from the same protein family (*Tc*AAAP), with calculated IC_50_ values in the range of 4.6–10.3 μM. It also showed a strong inhibition of trypomastigote burst from infected cells, with calculated IC_50_ of 130 nM (SI = 920) being significantly less effective on the epimastigote stage (IC_50_ = 30.6 μM). The effect of isotretinoin on the parasites plasma membrane permeability and on mammalian cell viability was tested, and no change was observed. Autophagosomes and apoptotic bodies were detected as part of the mechanisms of isotretinoin-induced death indicating that the inhibition of transporters by isotretinoin causes nutrient starvation that triggers autophagic and apoptotic processes. In conclusion, isotretinoin is a promising trypanocidal drug since it is a multi-target inhibitor of essential metabolites transporters, in addition to being an FDA-approved drug largely used in humans, which could reduce significantly the requirements for its possible application in the treatment of Chagas disease.

## Author summary

Polyamines are polycationic compounds essential for the regulation of cell growth and differentiation. In contrast with other protozoa, *Trypanosoma cruzi*, the etiological agent of Chagas disease, is auxotrophic for polyamines; therefore the intracellular availability of these molecules depends exclusively on transport processes. It was previously demonstrated that the lack of polyamines in *T*. *cruzi* leads to its death, making the polyamine transporter an excellent therapeutic target for Chagas disease. In this work, the polyamine permease *Tc*PAT12 was selected as a target for drug screening using 3000 FDA-approved compounds and computational simulation techniques. Using two combined virtual screening methods, isotretinoin, a well-known and safe drug used for acne treatment, bound to substrate recognition residues of *Tc*PAT12 and was chosen for further *in vitro* studies. Isotretinoin inhibited not only the polyamine transport but also all tested amino acid transporters from the same protein family as *Tc*PAT12. Interestingly, isotretinoin showed a high trypanocidal effect on trypomastigotes, with an IC_50_ in the nanomolar range. Autophagy and apoptosis were proposed as mechanisms of parasites death induced by isotretinoin. These results suggest that isotretinoin is a promising trypanocidal drug, being a multi-target inhibitor of essential metabolites transporters.

## Introduction

Chagas disease is a major health and economic problem in the Americas and its causative agent is the hemoflagellate *Trypanosoma cruzi* [1]. According to the World Health Organization, about 8 million people worldwide are infected with the parasite, and 10,000 people per year die from complications linked to Chagas disease, mostly in Latin America where the disease is endemic [2]. In addition, the chronicity of the pathology implies great health expenditures due to the disability associated with the chronic state of this infection, being heart failure the main disabling condition [3]. Only two drugs are approved for treating Chagas disease, the nitroimidazole benznidazole and the nitrofuran nifurtimox, which were discovered half a century ago and have very limited efficacy with severe side effects [4,5]. This highlights the need for the development of new therapeutic alternatives and the identification of novel drug targets.

Since transport of nutrients from the extracellular medium is inexpensive in terms of energy economy compared to their metabolic synthesis, the uptake is a very common and desirable strategy for parasitic organisms. *T*. *cruzi* is exposed to different environments along its life cycle, alternating between the gut of the insect vector, the bloodstream of the mammalian hosts, and within different cell types [6], and the availability of nutrients in these dissimilar milieus determines the need for complex metabolic adaptations. The first and probably the only multigenic family of amino acid transporters in *T*. *cruzi* (*Tc*AAAP) was identified by our group [7]. One interesting feature of these permeases is the absence of orthologs in mammalian genomes. Few members of this family have been characterized in trypanosomatids, including polyamines, arginine, proline and lysine permeases [8,9,10,11,12]. This *T*. *cruzi* transporter family comprises at least 36 genes coding for proteins with lengths of 400–500 amino acids and 10–12 predicted transmembrane α-helical spanners. Another remarkable feature of these proteins is the variability of the N-terminal domain (about 90 amino acids with only 5% of consensus positions), in contrast to the central and C-terminal domains, which have a very similar sequence [7,9]. In *Leishmania* spp. it was demonstrated that only the variable N-terminal domain is involved in the substrate specificity [13]. On the contrary, mutagenesis analysis in *T*. *cruzi* locates the substrate recognition site of the polyamine transporter outside the N-terminal variable region [14]. The high sequence similarity could be an advantage for the development of multi-target inhibitors against the *Tc*AAAP transporter family. The supply of essential polyamines in *T*. *cruzi* is exclusively achieved through transport processes, a clear case of metabolic-transport replacement in the evolutionary adaptation to parasitism [15]. Interestingly, *Tc*PAT12 (also known as *Tc*POT1) is the only polyamine transporter present in *T*. *cruzi*. Recent studies have shown that the trypanocidal drug pentamidine blocks *Tc*PAT12 in this parasite [16,17]. All this evidence highlights that *T*. *cruzi* nutrient transporters are promising targets for drug development. One interesting approach is the use of molecular docking to identify pharmacological active compounds among drugs already used for other therapeutic indications (called “drug repositioning” or “drug repurposing) [18]. For example, the discovery of polyamine analogs, by computational simulation, with inhibitory effects on the proliferation of *T. cruzi* has been recently reported [19].

Retinol (vitamin A, all-trans-retinol) and its derivatives play an essential role in metabolic functioning of the retina, the growth and differentiation of epithelial tissue, bone growth, reproduction, and immune response. Dietary retinol is derived from a variety of carotenoids found in plants, liver, egg yolks, and the fat component of dairy products. This compound activates retinoic acid receptors (RARs), inducing cell differentiation and apoptosis of some cancer cell types and inhibiting carcinogenesis [20,21,22,23,24]. Isotretinoin (13-cis-retinoic acid) is a retinol derivative used in the treatment of severe acne and some types of cancer [25,26]. The usage dose is 0.5–1 mg.kg^-1^ [27] and its most common side effects are skin xerosis, especially on exposed skin, cheilitis, telogen effluvium, inflammatory bowel disease and myalgia [28]. Despite its exact mechanism of action remains unknown, several studies have shown that this drug induces apoptosis in sebaceous gland cells. Isotretinoin has a low affinity for RARs and retinoid X receptors (RXR), but it may be intracellularly converted to metabolites that act as agonists of these nuclear receptors [29]. Previous data reported that butylated hydroxyanisole, retinoic acid and retinol acetate dramatically inhibit the growth of *Leishmania donovani* promastigotes, and retinol acetate also decreases by half the intracellular polyamine levels [30]. Furthermore, isotretinoin alters the life cycle of the protozoan parasite *Opalina ranarum* in frogs, inhibiting the induction of cyst formation [31]. Considering the effects of some retinoids in protozoan organisms, in this work we evaluated by virtual screening and *in vitro* assays different retinoids used in medical practice. We demonstrated that isotretinoin has trypanocidal effect through the specific inhibition of permeases from *Tc*AAAP family. As a repositioned drug, isotretinoin has many advantages over developing new drugs because of its oral bioavailability, low cost and current use for treating other diseases.

## Methods

### Virtual screening

**Fig 1:**
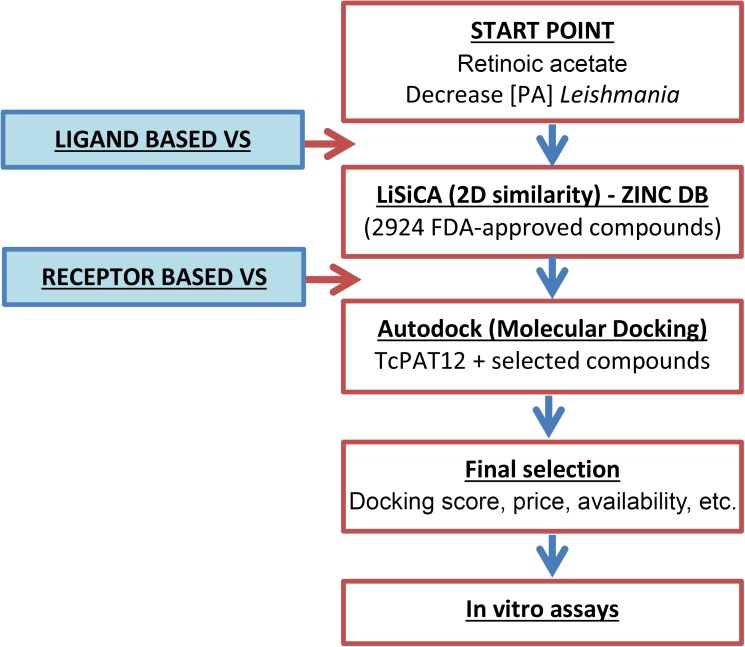
Workflow of the virtual screening strategy. A two-steps methodology was applied for identification of drug candidates to bind the *Tc*PAT12 substrate recognition site. The ligand-based virtual screening was performed using retinol acetate as reference molecule and a similarity searching algorithm based on the polynomial-time algorithm for finding maximal cliques (LiSiCA) [32]. Tested compounds were obtained from the ZINC database (http://zinc.docking.org/). The second step was a receptor-based strategy using the homology modeled transporter *Tc*PAT12 as the receptor, and selected compounds obtained from the similarity screening as putative ligands. Molecular docking was performed using the AutoDock software [34]. (PA, polyamines; VS, virtual screening).

Computational approaches for the identification of putative *Tc*PAT12 inhibitors started with a ligand-based virtual similarity screening search followed by molecular docking, which is a receptor-based technique. Retinol acetate was used as the reference compound for similarity searching in a database that comprises a total of 2924 worldwide commercially available drugs and nutraceuticals approved by U.S. Food and Drug Administration (FDA). This screening was performed using LiSiCA v1.0 (Ligand Similarity using Clique Algorithm) software [32], and similarities were expressed using the Tanimoto coefficient [33]. The structural data of compounds retrieved from similarity screening, as well as putrescine and spermidine, *Tc*PAT12 natural ligands. Selected molecules for molecular docking were obtained from a subset of the ZINC database (http://zinc.docking.org/). The compounds analyzed and their corresponding ZINC IDs were: acitretin (3798734), alitretinoin (12661824), etretinate (3830820), isotretinoin (3792789), putrescine (1532552), retinal (4228262), retinol (3831417), retinoic acid (12358651), retinol acetate (26892410), and spermidine (1532612). Further preparation of the PDBQT files (Protein Data Bank, partial charge (Q), and atom type (T)) was performed using AutoDock Tools v1.5.6 [34]. Three-dimensional structure of *Tc*PAT12 (GenBank ID: AY526253) was obtained by homology modeling, with as template the *Escherichia coli* amino acid antiporter (AdiC; PDB ID: 3L1L; about 30% amino acid identity with *Tc*PAT12) using the Swiss-Model server (http://swissmodel.expasy.org/) [35]. The model was refined using the software Modeller v7 and the previously reported model of the *Tc*PAT12 [14,36]. The obtained structure was evaluated by Ramachandran plotting using Chimera v1.8 [37,38]. From this model, residues Asn245, Tyr148 and Tyr400 were taken as flexible using AutoDock Tools 1.5.6. The grid parameter file was generated with Autogrid 4.2.6 so as to surround the flexible residues, with a grid map of 40 points in each dimension, a spacing of 0.0375 nm, and centered on position X = -2.794, Y = 9.659, and Z = 22.928. An additional docking assay was performed using a grid covering the whole transporter molecule, without defining specific flexible residues and using the same spacing and automatic centering. AutoDock 4.2.6 was used for calculation of optimal energy conformations for the ligands interacting with the protein active site, running the Lamarckian Genetic Algorithm 100 times for each ligand, with a population size of 300, and 2.7x10^4^ as the maximum number of generations. For each ligand, bound conformations were clustered and two criteria for selection of the preferred binding conformation were followed: taking the lowest free binding energy conformation of all the poses, and from the most populated cluster [39]. A diagram of the virtual screening workflow is shown in Fig 1.

### Parasites

Epimastigotes of *T*. *cruzi* Y strain (5x10^6^ cells.mL^-1^) were cultured at 28°C in plastic flasks (25 cm^2^), containing 5 mL of BHT (brain-heart infusion-tryptose) medium supplemented with 10% fetal calf serum, 100 U.mL^-1^ penicillin, 100 μg.mL^-1^ streptomycin and 20 μg.mL^-1^hemin [40]. CHO-K1 cells (Chinese Hamster Ovary) were cultured in RPMI medium supplemented with 10% heat-inactivated Fetal Calf Serum (FCS), 0.15% (w/v) NaCO_3_, 100 U/mL penicillin and 100 U/mL streptomycin at 37°C in 5% CO_2_. Trypomastigotes were obtained from the extracellular medium of CHO-K1 infected cells as previously described [41].

### Transport assays

Aliquots of epimastigote cultures (10^7^ parasites) were centrifuged at 8,000 xg for 30 s and washed once with phosphate-buffered saline (PBS). Cells were resuspended in 0.1 mL PBS and then 0.1 mL of the transport mixture containing the corresponding radiolabeled substrate was added: [^3^H]-putrescine, [^3^H]-proline, [^3^H]-lysine, [^3^H]-amino acids mixture (Ala, Arg, Asp, Glu, Gly, His, Ile, Leu, Lys, Phe, Pro, Ser, Thr, Tyr and Val), [^3^H]-thymidine or [^14^C]-glucose (PerkinElmer's NEN Radiochemicals; 0.4 μCi). Parasites were pre-incubated for 15 min with concentrations of isotretinoin between 0–50 μM for all molecules, except for putrescine in which case 0–100 μM were used. Following incubation at 28°C, the transport reaction was stopped by adding 1 mL of ice-cold PBS. Cells were centrifuged as indicated above and washed twice with ice-cold PBS. Cell pellets were resuspended in 0.2 mL of water and counted for radioactivity in UltimaGold XR liquid scintillation cocktail (Packard Instrument Co., Meridien CT, USA) [42,43]. Non-specific binding and carry over were evaluated by a standard transport assay supplemented with a 100-fold molar excess of the corresponding substrate.

### Trypanocidal activity assays

Exponentially growing *T*. *cruzi* epimastigotes were cultured as described above, in 24-wells plate at a start density of 10^7^ cells.mL^-1^ in BHT medium. Parasites growth was evaluated at different concentrations of isotretinoin, in the range of 0–300 μM, and parasite proliferation was determined after 72 h. Inhibition of trypomastigote bursting from infected cells was performed using CHO-K1 cells (5x10^4^ per well) infected with trypomastigotes (2.5×10^6^ per well) for 4 h. After this period, the infected cells were washed twice with PBS, the RPMI medium was replaced, and the cells were kept in culture in the presence of different concentrations of isotretinoin (0–30 μM) for 24 h. After infection, plates were incubated at 33°C and parasites were collected from the extracellular medium on the sixth day. Cells were counted using a Neubauer chamber using a blinded design or by viability assays using “Cell Titer 96 Aqueous One Solution Cell Proliferation Assay (MTS)” (Promega, Madison, WI, USA) according to the manufacturer instructions.

### Plasma membrane permeability assay

In order to test if isotretinoin exerts cell permeabilization, epimastigote cells (5x10^8^) were washed twice and resuspended in PBS. Aliquots of 100 μl containing 10^8^ parasites were mixed with 100 μl of the same buffer containing increasing amounts of isotretinoin (0, 5, 25 and 100 μM). After 30 min of incubation at room temperature in the presence of isotretinoin, the tubes were centrifuged at 16100 xg for 2 min. Supernatants were kept on ice and pellets were resuspended in the same buffer. Permeabilization of epimastigotes with digitonin was used as a positive control; cells were washed twice and resuspended in 50 mM Tris-HCl buffer, pH 7.5, containing 0.25 M sucrose and 10 μM E64. Aliquots of 100 μl containing 10^8^ parasites were mixed with 100 μl of the same buffer containing 0.3 mg.mL^-1^ of digitonin. After 2.5 min of incubation at room temperature, the tubes were centrifuged at 16100 ×g for 2 min. Supernatants were transferred to new tubes and pellets were resuspended in the same buffer. All supernatant and pellet fractions were analyzed by Western blot. Briefly, samples were run on 15% SDS-polyacrylamide gels (PAGE) and transferred onto a PVDF membrane. The membranes were blocked and incubated with primary rabbit antibodies anti-glutamate dehydrogenase (1:5000 dilution) followed by incubation with peroxidase-conjugated anti-rabbit (1:5000 dilution). The peroxidase-labeled proteins were revealed using Super Signal West Pico Chemiluminescent substrate following the manufacturer instructions (Pierce, Waltham, MA, USA).

### Determination of epimastigotes death mechanisms

For apoptosis analysis by TUNEL (Terminal deoxynucleotidyl transferase dUTP nick end labeling), parasites (10^7^) were treated with the corresponding concentrations of isotretinoin and, after letting the cells settle for 20 min onto poly-L-lysine coated coverslips, were fixed for 20 min with 4% paraformaldehyde in PBS and permeabilized with 0.1% Triton X-100. Assays were performed using the “In situ cell death detection Kit” (Roche) according to the manufacturer instructions. Positive and negative controls were made using DNAse I and untreated parasites, respectively. Slides were mounted using Vectashield with DAPI (Vector Laboratories) and cells were observed under an Olympus BX60 fluorescence microscope. Images were recorded with an Olympus XM10 camera. To detect phosphatidylserine, annexin V binding on the external surface of the plasma membrane of treated and untreated parasites was evaluated using the “Annexin V: FITC Apoptosis Detection Kit” (Sigma-Aldrich) according to the manufacturer’s protocol. Co-staining of the parasites with propidium iodide was performed to evaluate the integrity of plasma membrane during the treatments. Fluorescence was detected in FACSCalibur equipment (Becton Dickinson & Co., NJ, USA). Data was analyzed using Cyflogic software. [44]. Autophagy was evaluated using monodansylcadaverine (MDC) labeling [45]. Briefly, after isotretinoin treatment, parasites were incubated with 0.05 mM MDC in PBS at 37°C for 15 min and washed twice in PBS. MDC stain was evaluated using a fluorescence microscope Olympus BX60 and images were captured with an Olympus XM10 digital camera. To evaluate the formation of autophagic structures by indirect immunoflurescence microscopy, epimastigote samples were collected, washed twice with PBS, and settled for 20 min at room temperature onto poly L-lysine coated coverslips. Parasites were then fixed at room temperature for 20 min with 4% formaldehyde in PBS, permeabilized with cold methanol for 5 min, and rehydrated in PBS for 15 min. The samples were blocked with 1% BSA in PBS for 10 min and incubated with the primary antibody in blocking buffer (rabbit anti-Atg8.1 polyclonal, 1:250 dilution) for 2 h. The antibody was kindly provided by Dra. Vanina E. Alvarez from the “Instituto de Investigaciones Biotecnológicas” (IIB-INTECH). After three washes, the parasites were incubated with anti-rabbit antibodies tagged with FITC, at a dilution of 1:500 for 30 min, washed and mounted using Vectashield with DAPI (Vector Laboratories). Cells were observed in an Olympus BX60 fluorescence microscope and recorded with an Olympus XM10 camera. To detect apoptotic bodies, cultures were stained with acridine orange and ethidium bromide. The morphology of death and surviving cells were observed by fluorescence microscopy. Ethidium bromide only enters into non-viable cells and stains chromatin and apoptotic bodies with an orange color. Acridine orange penetrates in viable cells and turns green when it intercalates with DNA [46].

### Statistics and data analysis

All the experiments were made at least in triplicates and results presented here are representative of three independent assays. IC_50_ values were obtained by non-linear regression of dose-response logistic functions, using GraphPad Prism 6.01.

## Results

### Virtual screening studies

**Fig 2:**
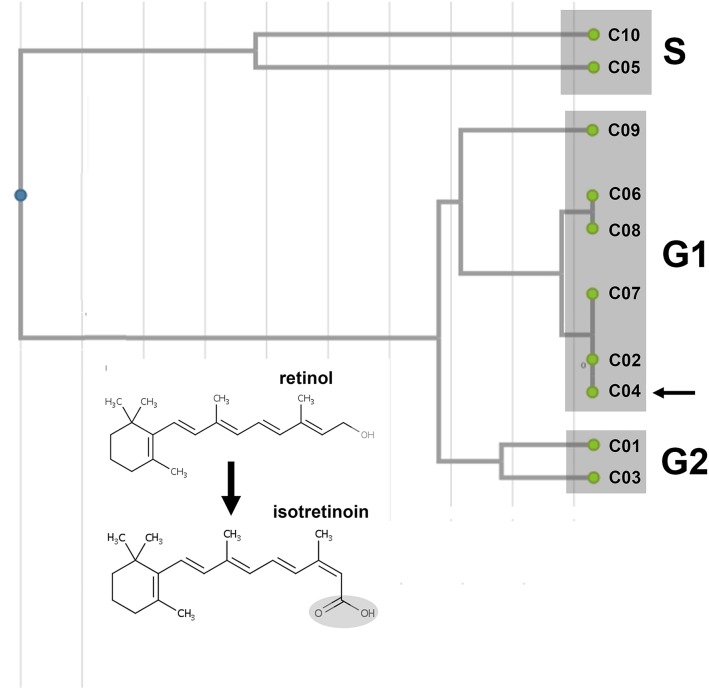
Similarity dendrogram of the selected compounds. Seven therapeutic retinoids obtained in the first step of virtual screening were used to construct a dendrogram from a similarity matrix, based on the Tanimoto coefficient using the Unweighted Pair Group Method with Arithmetic mean (UPGMA) algorithm [33]. The different generations of retinoids (G1 and G2) and the natural substrates of *Tc*PAT12 (S; putrescine and spermidine) were indicated in the different clusters. Numbers are referred to the compounds listed in Table 1. The arrow indicates the selected compound, isotretinoin. The inset shows the structures of retinol and its derivative isotretinoin.

Two consecutive virtual screening techniques were applied to find out putative *Tc*PAT12 inhibitors. As mentioned, previous data demonstrated that retinol acetate has toxic effects on *Leishmania* parasites by diminishing the intracellular polyamine concentrations [30]. According to these results, this molecule was used as a reference compound for virtual screening. In order to get more ligands to test besides retinol acetate, the first approach was a ligand-based virtual screening using a database containing 2924 FDA approved drugs and nutraceuticals. Seven therapeutic retinoids [47] were obtained from the first step of virtual screening and were used to construct a similarity matrix (S1 Fig) and a dendrogram based on the Tanimoto coefficient, using the Unweighted Pair Group Method with Arithmetic mean (UPGMA) algorithm [33,48]. The similarity graphic discriminates between clusters containing the different generations of retinoids which are structurally unrelated to the natural substrates of *Tc*PAT12 (Fig 2).

**Fig 3:**
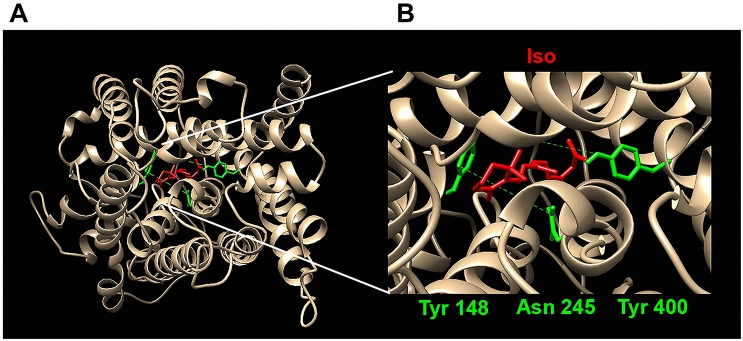
Predicted binding between isotretinoin and *Tc*PAT12. (A) The three-dimensional model of *Tc*PAT12 (GenBank ID: AY526253) was obtained by homology modeling in the Swiss-model server [35] using the *Escherichia coli* amino acid antiporter (PDB ID: 3L1L) as a template and refined using a previously reported modeled structure [14]. Residues taken as flexible for docking analysis are indicated in green and the ligand isotretinoin in red. (B) Detailed docked conformation of isotretinoin inside the binding site of homology modeled *Tc*PAT12. The figure shows the residues where the substrate putrescine and isotretinoin bind to the transporter.

The second step was a receptor-based strategy using molecular docking simulations. The three-dimensional structure of *Tc*PAT12 was modeled using as a template the *Escherichia coli* amino acid antiporter, and refined with experimentally validated data about the putrescine binding site [14]. Since evaluation methods for homology models quality were made based on the data available on the PDB, they are biased towards globular proteins, and cannot be used for membrane proteins [49]. For that reason, the quality of the *Tc*PAT12 model was evaluated by checking torsion angles of the peptide backbone in a Ramachandran Plot, one of the most powerful tools used to determinate the quality of a model coordinates [37,50] (S2 Fig). Results showed that the obtained model has only 4.3% of the torsions in the outlier regions of the plot, and none of those residues were involved in the active site of the transporter. Given that 91% of the experimental structures deposited in the PDB have 10% or less residues in the outlier region, and only 76.5% possess less than 5% of outliers, the generated model for *Tc*PAT12 can be considered to have a reasonable quality [51]. The ability of the selected retinoids to interact with *Tc*PAT12 substrate binding residues was tested by a computer-assisted simulation with AutoDock 4.0, using the natural substrates of *Tc*PAT12 as binding parameter references (Table 1). For each compound, two criteria were used to analyze docking results: the lowest global binding score, and the lowest binding score from the most populated cluster. Isotretinoin had the lowest binding energy values for both sorting criteria. These results, together with its availability and market price, point it out as the best candidate for further analysis. According to docking models, isotretinoin binds within the hydrophobic channel of the transporter, in the previously reported putrescine-binding pocket, interacting with residues Asn245, Tyr148 and Tyr400 (Fig 3A and 3B).

**Table 1:** Compounds obtained by similarity screening and molecular docking analysis.

#	Compound (popular name)	Compound (brand name)	ΔG (kcal.mol^-1^) (all)	ΔG (kcal.mol^-1^) (MPC)
C1	Acitretin	Neotigason	-6.70	-6.70
C2	Alitretinoin	Panretin	-9.56	-9.56
C3	Etretinate	Tegison	+4.13	+4.13
**C4**	**Isotretinoin**	**Roaccutan**	**-10.78**	**-10.78**
C5	Putrescine*	-	-3.31	-3.31
C6	Retinal	-	-8.86	-8.86
C7	Retinoic acid	Atralin	-9.07	-9.07
C8	Retinol	-	-8.69	-8.69
C9	Retinol acetate	-	-10.02	-9.23
C10	Spermidine*	-	-3.08	-3.08

All the retinoids obtained by similarity searching, in addition to the reference compounds and the substrates of *Tc*PAT12 (*), were listed in the table. Columns indicate the compound abbreviation (#), the popular name and one of the brand names obtained from the zinc database (http://zinc.docking.org/), the lowest ligand efficiency (ΔG all), and the lowest ligand efficiency of the most populated cluster (ΔG MPC). Ligand efficiency values were calculated using the AutoDock software. Dash (-) instead of brand name indicates that the compound is marketed under its chemical (popular) name.

Isotretinoin possess a ligand efficiency of -0.37 kcal.mol^-1^ for its interaction with the polyamine binding site of *Tc*PAT12, and the cluster with the lowest global free binding score (-10.78 kcal.mol^-1^) was also the one with more conformations, with 35 of the 100 generated poses. Interestingly, when docking a simulation between *Tc*PAT12 and isotretinoin was performed over the whole transporter molecule, without limiting the region to be tested, similar results were obtained; isotretinoin also bound residues Asn245, Tyr148, and Tyr400. Both results suggest that isotretinoin binds more stably in that region of *Tc*PAT12 than its natural ligands. All these data are summarized in Table 1.

### Effect of isotretinoin and other retinoids on polyamine transport

**Fig 4:**
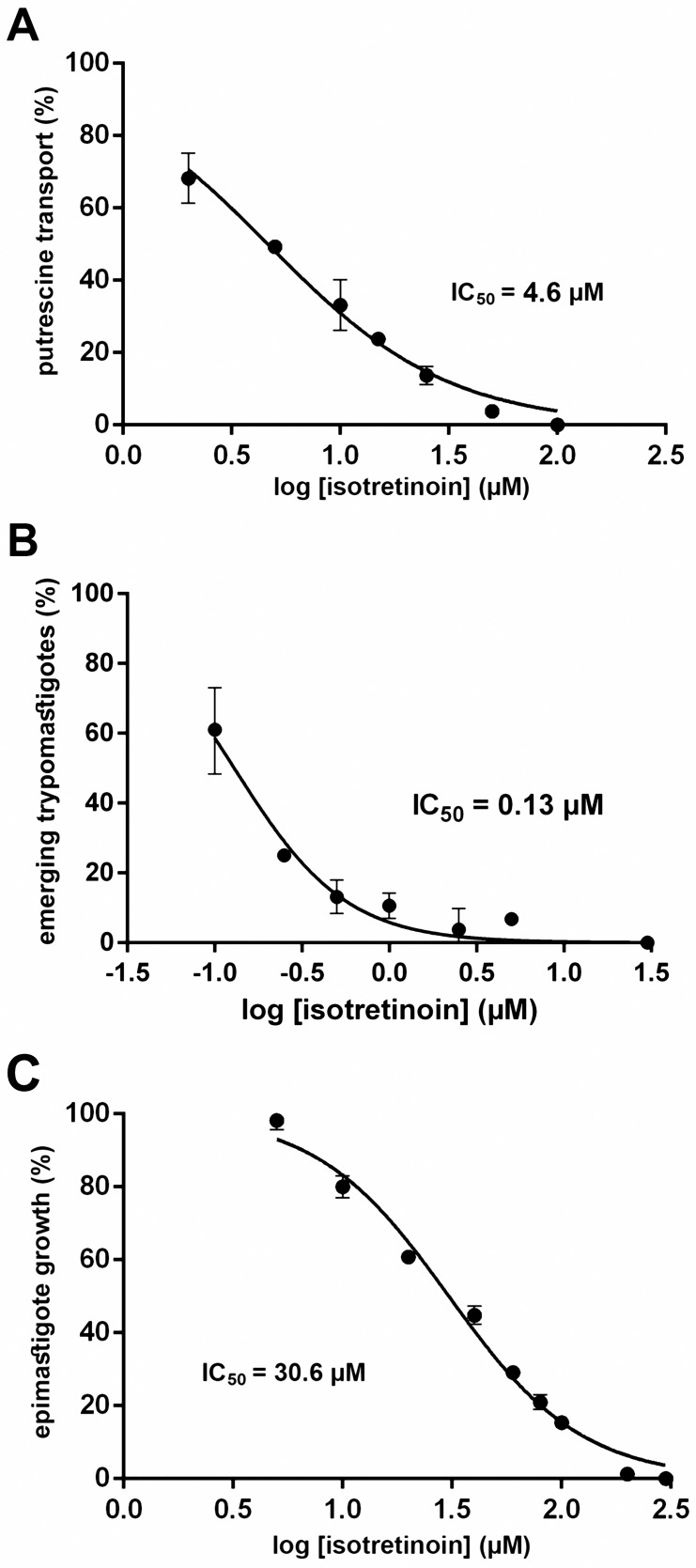
Effect of isotretinoin on polyamine transport and parasites growth. (A) Putrescine transport was measured using epimastigote cultures (10^7^ parasites) and 100 μM [^3^H]-putrescine, as indicated under Methods, in presence of isotretinoin 0–100 μM (plotted in log scale). Transport values are given as the percentage of the transport rate in untreated parasites (11.7 pmol.min^-1^). The IC_50_ value was calculated by non-linear regression using the GraphPad Prism software. (B) CHO-K1 cells were infected with trypomastigote forms (in a proportion 1:50) for 4 h. Infected cells were treated with different concentrations of isotretinoin between 0 and 30 μM for 24 h. After treatment, plates were incubated at 33°C and trypomastigotes were collected from the extracellular medium on the sixth day. Parasite density is expressed as the percentage of untreated parasites (1.23 x10^6^ cells.mL^-1^). (C) 10^7^ exponential phase epimastigotes were treated with different concentrations of isotretinoin between 0 and 300 μM for 72 h. Cell density is expressed as the percentage of untreated parasites (6.6x10^7^ cells.mL^-1^). All the IC50 calculations were carried as described in “Trypanocidal activity assays” under the Methods section.

In order to validate the results obtained by virtual screening, the ability of isotretinoin to inhibit putrescine uptake through *Tc*PAT12 was evaluated. Putrescine concentration was fixed in 100 μM, about 10-fold its *K*m value [52], and transport assays were performed in the presence of different concentrations of isotretinoin in the range of 0–100 μM. Results shown in Fig 4A confirmed that low concentrations of this drug produce a significant inhibition of putrescine transport. The calculated isotretinoin concentration that inhibited 50% of the putrescine transport (IC_50_) was 4.6 μM. To evaluate if the putrescine transport could be inhibited by other retinoids that scored promising ΔG values in the docking assays, the experiments were repeated with acitretin (ΔG = -6.70 kcal.mol^-1^), a drug used for psoriasis treatment, and the isotretinoin precursor retinol (ΔG = -8.69 kcal.mol^-1^). The IC_50_ of acitretin was 6.8 μM while retinol had no effect on putrescine transport in the tested concentrations.

### Effect of isotretinoin on other transport systems

The amino acid and polyamine transporters of the *Tc*AAAP family are very similar in terms of amino acid sequences [7]. For this reason, the inhibitory effect of isotretinoin on other transporters from the same family, and also over structurally unrelated permeases, was evaluated. IC_50_ values were calculated with the same criteria used for putrescine transport; about 10-fold the *K*m concentration of each compound, in the presence of isotretinoin from 0 to 50 μM. The assayed substrates of *Tc*AAAP transporters were proline, lysine, and an amino acid mix [9,12], while thymidine and glucose incorporation was evaluated for effect of isotretinoin on unrelated permeases [53]. Calculated IC_50_ values for proline, lysine and the amino acid mix were 10.3 μM, 5.1 μM and 5.8 μM, respectively. On the other hand, isotretinoin produced no significant inhibition on thymidine and glucose transport demonstrating its specificity for members of the *Tc*AAAP family.

### Evaluation of isotretinoin trypanocidal activity

With the aim of analyzing if the inhibition of putrescine uptake could affect the parasites viability, isotretinoin toxicity over trypomastigotes and epimastigotes was assessed. The effect of isotretinoin in trypomastigotes, the mammal infective form of *T*. *cruzi*, was evaluated using a model of *in vitro* infection in CHO-K1 cells. Infected cells were exposed to isotretinoin for 24 h in the concentration range of 0–30 μM. At very low concentrations, isotretinoin inhibited the trypomastigotes burst after six days of infection, with a calculated IC_50_ of 130 nM (Fig 4B). Remarkably, this IC_50_ value is significantly lower than those obtained for the drugs currently used as a treatment for Chagas disease [12,16]. The calculated selectivity index of isotretinoin against trypomastigotes over human macrophages was about 920. In addition, the infection index (infected cells x total amastigotes per cell) was calculated for infected cells treated with isotretinoin from 65 to 260 nM. For control cells it was 6.44 (±1.55) and for cells treated with 65, 130 and 260 nM were 6.49 (±1.98), 4.13 (±1.16) and 3.61 (±0.94), respectively. Epimastigotes, the insect stage of *T*. *cruzi*, were also treated with different concentrations of isotretinoin (0–300 μM) for 72 h. As Fig 4C shows, isotretinoin was also effective as growth inhibitor of cultured epimastigotes, but at concentrations 230-fold higher than in trypomastigotes (IC_50_ = 30.6 μM).

To evaluate the cytotoxicity of isotretinoin on CHO-K1 cells and peripheral blood monocytes, cells were exposed to isotretinoin for 24 h in a concentration range from 0 to 30 μM, and no effect was observed at any of these drug concentrations.

### Determination of isotretinoin trypanocidal mechanism

**Fig 5:**
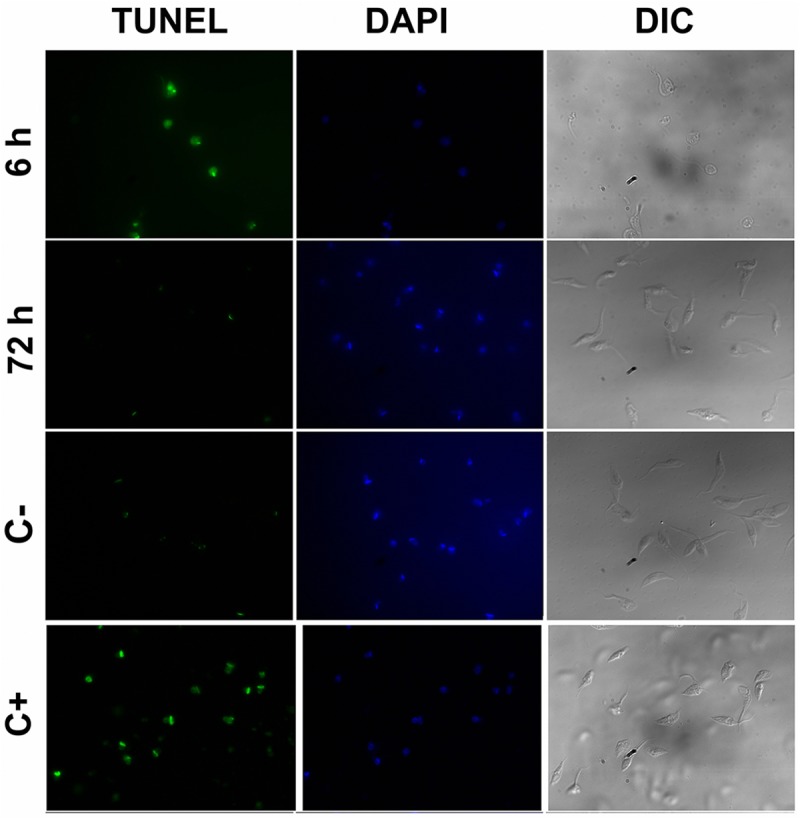
Fluorescence micrographs of TUNEL-stained epimastigotes. Epimastigote cells were treated with DNAse I (apoptosis positive control, C+), without treatment (negative control, C-) or 200 and 30 μM isotretinoin for 6 and 72 h, respectively. Parasites were fixed and permeabilized after the TUNEL reaction (green) and stained with DAPI (blue). Images acquired by fluorescence microscopy under 100x objective showed positive TUNEL in DNAse I control and cells treated with 200 μM isotretinoin for 6 h. Differential interference contrast (DIC) images showed the differences in cell morphology (partially or fully rounded cells) after 6 h isotretinoin exposure. The TUNEL protocol was detailed under Methods.

**Fig 6:**
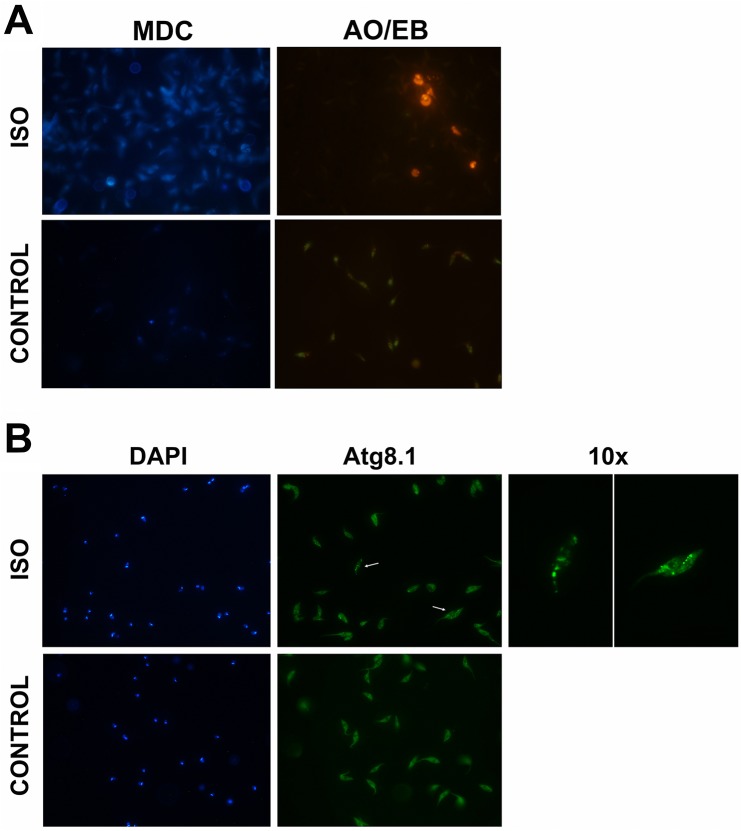
Fluorescence microscopy analysis of autophagic vacuoles and apoptotic bodies in epimastigotes treated with isotretinoin. ** (A)** Parasites were treated with 200 μM isotretinoin for 6 h (ISO) and without the drug (negative control, CONTROL). After this period, cells were evaluated with MDC to detect autophagosomes. Cells treated with the drug showed fluorescent probe (blue) accumulated in autophagic vacuoles. To identify apoptosis, parasites with the same treatment conditions were stained with acridine orange and ethidium bromide (AO/EB). Apoptotic bodies were detected only in the treated cells (orange). **(B)** Analysis of autophagic structures. Immunofluorescence microscopy of untreated parasites (CONTROL) and exposed to 200 μM isotretinoin for 6 h (ISO), marked with anti-Atg8.1 antibody. Atg8.1 protein is a autophagosomal membrane marker. The arrows indicate the parasites that were magnified (10x) in the images on the right. The nuclei and kinetoplasts were stained with DAPI (blue).

In order to test if the observed inhibition of parasites growth was mediated by a programmed cell death mechanism, apoptosis analysis in epimastigote cells was performed. The first approach was to evaluate the exposition of phosphatidylserine (annexin V) and propidium iodide exclusion by flow cytometry. As S3 Fig shows both cell death markers were negative in epimastigotes exposed to isotretinoin from 30 to 120 μM for 72 h. The second technique used was the TUNEL assay. Epimastigote cells treated with 30 μM isotretinoin for 72 h presented a TUNEL negative staining (Fig 5). In order to evaluate apoptosis using a short-time isotretinoin treatment, the IC50 for epimastigotes was calculated at 6 h post-treatment, with a value of 214 μM. At 200 μM isotretinoin, the percentage of TUNEL positive cells decreased to 64.9% (± 0.03) showing a complete change in cell morphology, partially or fully rounded cells. Negative and positive controls were also assayed, using untreated cells and cells treated with DNAse I, respectively. Under these conditions the exposition of phosphatidylserine and propidium iodide were also evaluated. Flow cytometry analysis showed that 21.3% of the parasites treated with 200 μM drug were positive for annexin V and all the cells population was negative to propidium iodide. These results suggest that parasites entered only in apoptosis, no necrosis process was observed (S3 Fig, lower panel). In addition, to assess whether an autophagic component is involved in cell death induced by isotretinoin treatment, parasites were evaluated using MDC, a fluorescent probe that accumulates in autophagic vacuoles [45]. Parasites treated for 6 h with 200 μM isotretinoin presented rounded structures stained by MDC, (Fig 6A). To validate the formation of autophagic structures, subcellular localization of *Tc*Atg8.1 protein, an autophagosomal membrane marker [54], was evaluated by indirect immunofluorescence microscopy. Autophagic vacuoles were detected in parasites also treated for 6 h with 200 μM isotretinoin, indicating that autophagic processes were triggered by this drug (Fig 6B). Finally, when parasites under the same treatment conditions were stained with ethidium bromide and acridine orange, apoptotic bodies were detected (Fig 6A).

### Effect of isotretinoin on the stability of the plasma membrane

To determine if the trypanocidal effect of isotretinoin was due to an increased permeability of the plasma membrane, permeabilization experiments with this drug were carried out using the non-ionic detergent digitonin as a positive control (S4 Fig). The pattern of extraction of glutamate dehydrogenase, which localizes in the parasites cytosol, was used as a membrane stability marker [55]. At isotretinoin concentrations up to 100 μM, Western Blot analysis showed that glutamate dehydrogenase was completely absent in the parasites supernatant demonstrating that the structure of the plasma membrane remained unaltered at these drug concentrations. Positive control experiments using digitonin 0.3 mg.mL^-1^ showed that the cytosolic marker had been totally extracted. The plasma membrane integrity was also evaluated by propidium iodide exclusion using flow cytometry. Results of treatments up to 120 μM for 72 h and 200 μM for 6 h, suggest that the plasma membrane remains unaltered after isotretinoin exposure (S3 Fig).

## Discussion

Two of the most promising alternatives related to the Chagas disease therapy were the use of benznidazole in the chronic phase of the disease (BENEFIT) and the implementation of posaconazole as a novel trypanocidal drug. Unfortunately, after a recent evaluation of their effectiveness, none of these alternatives was successful [56,57] highlighting the urgent need for the development of new therapeutic substitutes for conventional treatments. In this context, drug repositioning is a rapid way to obtain compounds with new desired biological activity from drugs already approved for human use, smoothing the path for quickly reaching the counters [58]. Within *in silico* strategies for drugs identification, the combination of different virtual screening techniques significantly enhances the possibility to succeed in subsequent *in vitro* and *in vivo* studies [18]. In this work the strategy used was a combination of a ligand-based virtual screening by similarity followed by a receptor-based technique (molecular docking). The *T*. *cruzi* polyamine permease *Tc*PAT12 is a promising therapeutic target for rational drug design since this parasite is the only trypanosomatid unable to synthesize polyamines *de novo*, which makes it dependent on transport processes [59]. Besides, as reported so far, this is the only polyamine transporter present in *T*. *cruzi*.

Isotretinoin was selected on the basis of its low predicted free binding energy and for being a very common, low-cost compound. Isotretinoin is a retinol derivative, naturally found in small quantities in the human body and mainly used in the treatment of severe acne [60]. Another advantage is its market price of about USD 300–400 per kilogram which would make it easily accessible for developing countries. The calculated lowest free binding energy of isotretinoin to *Tc*PAT12 substrate recognition site was -10.78 kcal.mol^-1^. This value is similar to the obtained using the specific human polyamine transport blocker AMXT-1501 (-14.01 kcal.mol^-1^) [61] and lower than those of the natural substrates, putrescine (-3.31 kcal.mol^-1^) and spermidine (-3.08 kcal.mol^-1^). These data suggest that the stability of the isotretinoin—transporter complex is higher than those formed with its natural substrates probably because of the greater number of atoms (23) capable of engage in molecular interactions. When three of the molecules predicted to bind to the transporter were tested, isotretinoin and acitretin produced a strong inhibition of putrescine transport, while retinol had no effect. This is due to the predictability achievable by AutoDock simulations, which because of the completely theoretical nature of their scoring function, when compared with experimental models of other ligand-membrane protein interactions, have demonstrated 62% chances of identifying active compounds, while 44% chances of misidentifying an inactive compound as an active one [62]. Isotretinoin inhibition of transport correlated with its trypanocidal activity in epimastigotes. The IC_50_ calculated for trypomastigotes was in the nanomolar order and about 230-fold higher than the observed in epimastigotes, and also had a value similar to that of a new proteasome inhibitor tested by Novartis for treating *T*. *cruzi* infection in mice [63]. When compared, the effect of the drug in trypomastigotes was almost three orders of magnitude higher than its effect in human macrophages (selectivity index). These results are important since only the mammalian *T*. *cruzi* stages are relevant from a therapeutic perspective. In addition, the concentration at which isotretinoin acts *in vitro* is one order of magnitude lower than those reported for the drugs currently used for the treatment of Chagas disease; benznidazole and nifurtimox [12,16]. Isotretinoin is a good candidate for the treatment of Chagas disease since it does not require to be chemically modified. This feature is relevant since after any chemical modification it will be considered as a new drug and not as a repositioned one, with the consequent expensive trials required for its approval. Once the effect of isotretinoin over *Tc*PAT12 was validated, inhibition of other amino acid transporters from the same family was tested. Remarkably, isotretinoin inhibited all the assessed transporters from *Tc*AAAP family and no effect was observed over structurally unrelated proteins such as hexoses and nucleosides transporters. This specificity could be explained by the high structural similarity between all members of polyamines and amino acids transporters of the *Tc*AAAP family.

Autophagy is a mechanism by which cells under starvation digest their own components to provide amino acids that may function as an energy source [44] and this process was reported in trypanosomatids more than 10 years ago [64]. Considering that isotretinoin inhibited polyamine and also amino acid transporters, the consequent nutrient starvation would initiate autophagic process that might not recover the cells and thus the programmed cell death by apoptosis might be triggered. This is particularly relevant in the case of *T*. *cruzi* since epimastigotes use amino acids as the main carbon and energy sources alternative to glucose, as well as the use of amino acids in stage differentiation, host cells invasion, stress resistance, cell energy management, among others [12,65,66,67]. Isotretinoin acts as a multi-target inhibitor of transporter proteins from the *Tc*AAAP family, improving its trypanocidal potential as well as diminishing the possibility of generating drug resistance in the parasite. In addition, isotretinoin probably acts on the transporters in the external side of the plasma membrane, avoiding one of the most common problems of drugs, such as the way of entry into the cells.

Summarizing, isotretinoin is a promising trypanocidal drug because it has activity in the nanomolar range of concentrations, it is a multi-target inhibitor of essential metabolites transporters, it is already approved by the FDA and also it is a drug largely used in humans, which significantly reduces the requirements for its application in therapy for Chagas disease.

## Supporting information

S1 Fig Similarity matrix, based on the Tanimoto coefficient. All compounds (C1 –C10) obtained from the first step of virtual screening were used to construct a similarity matrix. Indicated values were calculated based on the Tanimoto coefficient by comparing between each set of similarity scores. The name of each compound was indicated in Table 1.
(PDF)

S2 Fig Evaluation of *Tc*PAT12 model by Ramachandran Plot. Analysis of Φ and Ψ backbone angles on a Ramachandran plot for the homology modeled *Tc*PAT12, with 86.5% of residues in the favored region, 9.1% in allowed region and 4.3% in outlier region.
(PDF)

S3 Fig Apoptosis and membrane stability analysis. Dot-plot graphics of flow cytometry analysis of annexin-V labeling (x-axis) and propidium iodide (y-axis) were performed in order to evaluate apoptosis and the plasma membrane permeability of parasites treated with 30, 60 and 120 μM of isotretinoin for 72 h (upper panel), 200 μM of drug for 6 h (lower panel) and untreated cells (control). The results suggest that the plasma membrane remains unaltered after isotretinoin exposure and necrotic processes were not observed in any treatment. Only the parasites exposed to 200 μM for 6 h were positive for annexin V.
(PDF)

S4 Fig Effect of isotretinoin on the plasma membrane. Extraction experiments were carried out using isotretinoin (A) or the non-ionic detergent digitonin as extraction control (B). 10^8^ parasites were incubated with increasing amounts of isotretinoin (5, 25 and 100 μM), or digitonin (0 and 0.3 mg.mL^-1^). Fractionation results were analyzed by Western blot using antibodies anti- *T*. *cruzi* glutamate dehydrogenase which localizes in the parasites cytosol. Upper and lower lines of each figure showed the pellet and supernatant fractions after extraction, respectively. Numbers above each figure indicate the concentration of isotretinoin (A) or digitonin (B). The detailed protocol was described under Methods.
(PDF)
