# Supplementary material for: Trypanocidal Effect of Isotretinoin through the Inhibition of Polyamine and Amino Acid Transporters in Trypanosoma cruzi
Source: PLoS Negl Trop Dis. 2017 Mar 17;11(3):e0005472. doi: 10.1371/journal.pntd.0005472 (PMC5371382; doi:10.1371/journal.pntd.0005472)
Supplement: S1 Fig — All compounds (C1 –C10) obtained from the first step of virtual screening were used to construct a similarity matrix. Indicated values were calculated based on the Tanimoto coefficient by comparing between each set of similarity scores. The name of each compound was indicated in Table 1. (PDF) [file pntd.0005472.s001.pdf]

	C1	C2	C3	C4	C5	C6	C7	C8	C9	C10
C1	1	0,84	0,85	0,84	0,8	0,8	0,84	0,66	0,13	0,15
C2	0,84	1	0,71	1	0,95	0,95	1	0,77	0,14	0,17
C3	0,85	0,71	1	0,71	0,74	0,74	0,71	0,79	0,13	0,14
C4	0,84	1	0,71	1	0,95	0,95	1	0,77	0,14	0,17
C5	0,8	0,95	0,74	0,95	1	1	0,95	0,8	0,15	0,17
C6	0,8	0,95	0,74	0,95	1	1	0,95	0,8	0,15	0,17
C7	0,84	1	0,71	1	0,95	0,95	1	0,77	0,14	0,17
C8	0,66	0,77	0,79	0,77	0,8	0,8	0,77	1	0,13	0,15
C9	0,13	0,14	0,13	0,14	0,15	0,15	0,14	0,13	1	0,45
C10	0,15	0,17	0,14	0,17	0,17	0,17	0,17	0,15	0,45	1

S1 Fig. Similarity matrix, based on the Tanimoto coefficient. All compounds (C1 – C10), obtained from the first step of virtual screening, were used to construct a similarity matrix. Indicated values were calculated based on the Tanimoto coefficient by comparing between each set of similarity scores. The name of each compound was indicated in Table 1.
