# Supplementary material for: Trypanocidal Effect of Isotretinoin through the Inhibition of Polyamine and Amino Acid Transporters in Trypanosoma cruzi
Source: PLoS Negl Trop Dis. 2017 Mar 17;11(3):e0005472. doi: 10.1371/journal.pntd.0005472 (PMC5371382; doi:10.1371/journal.pntd.0005472)
Supplement: S2 Fig — Analysis of Φ and Ψ backbone angles on a Ramachandran plot for the homology modeled TcPAT12, with 86.5% of residues in the favored region, 9.1% in allowed region and 4.3% in outlier region. (PDF) [file pntd.0005472.s002.pdf]

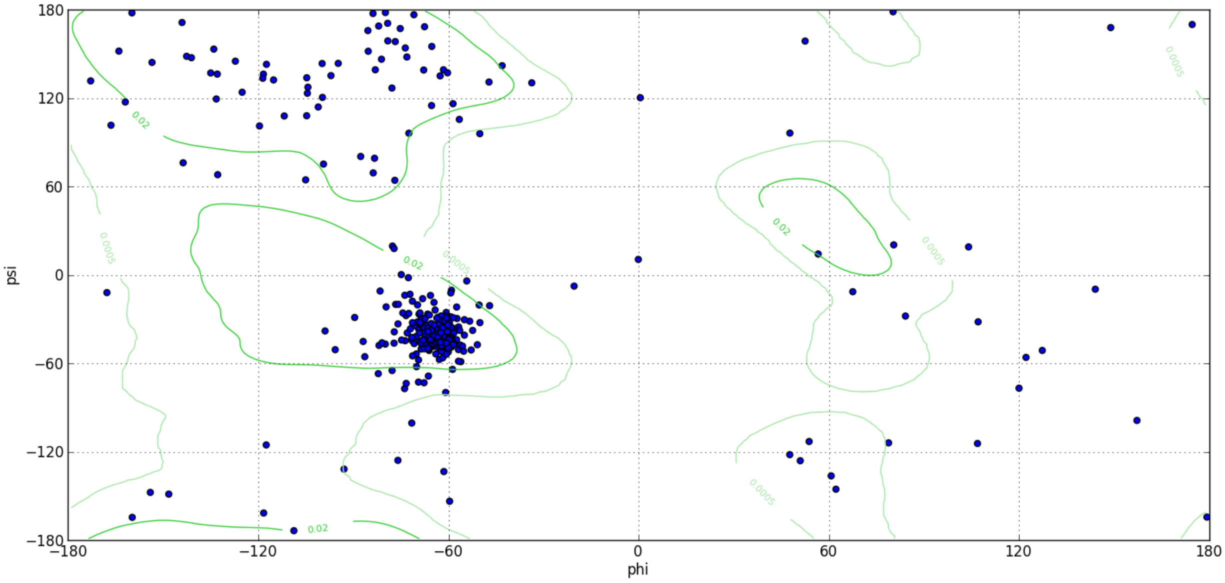

S2 Fig. Evaluation of *Tc*PAT12 model by Ramachandran Plot. Analysis of Φ and Ψ backbone angles on a Ramachandran plot for the homology modeled *Tc*PAT12, with 86.5% of residues in the favored region, 9.1% in allowed region and 4.3% in outlier region.
