# Supplementary material for: Trypanocidal Effect of Isotretinoin through the Inhibition of Polyamine and Amino Acid Transporters in Trypanosoma cruzi
Source: PLoS Negl Trop Dis. 2017 Mar 17;11(3):e0005472. doi: 10.1371/journal.pntd.0005472 (PMC5371382; doi:10.1371/journal.pntd.0005472)
Supplement: S3 Fig — Dot-plot graphics of flow cytometry analysis of annexin-V labeling (x-axis) and propidium iodide (y-axis) were performed in order to evaluate apoptosis and the plasma membrane permeability of parasites treated with 30, 60 and 120 μM of isotretinoin for 72 h (upper panel), 200 μM of drug for 6 h (lower panel) and untreated cells (control). The results suggest that the plasma membrane remains unaltered after isotretinoin exposure and necrotic processes were not observed in any treatment. Only the parasites exposed to 200 μM for 6 h were positive for annexin V. (PDF) [file pntd.0005472.s003.pdf]

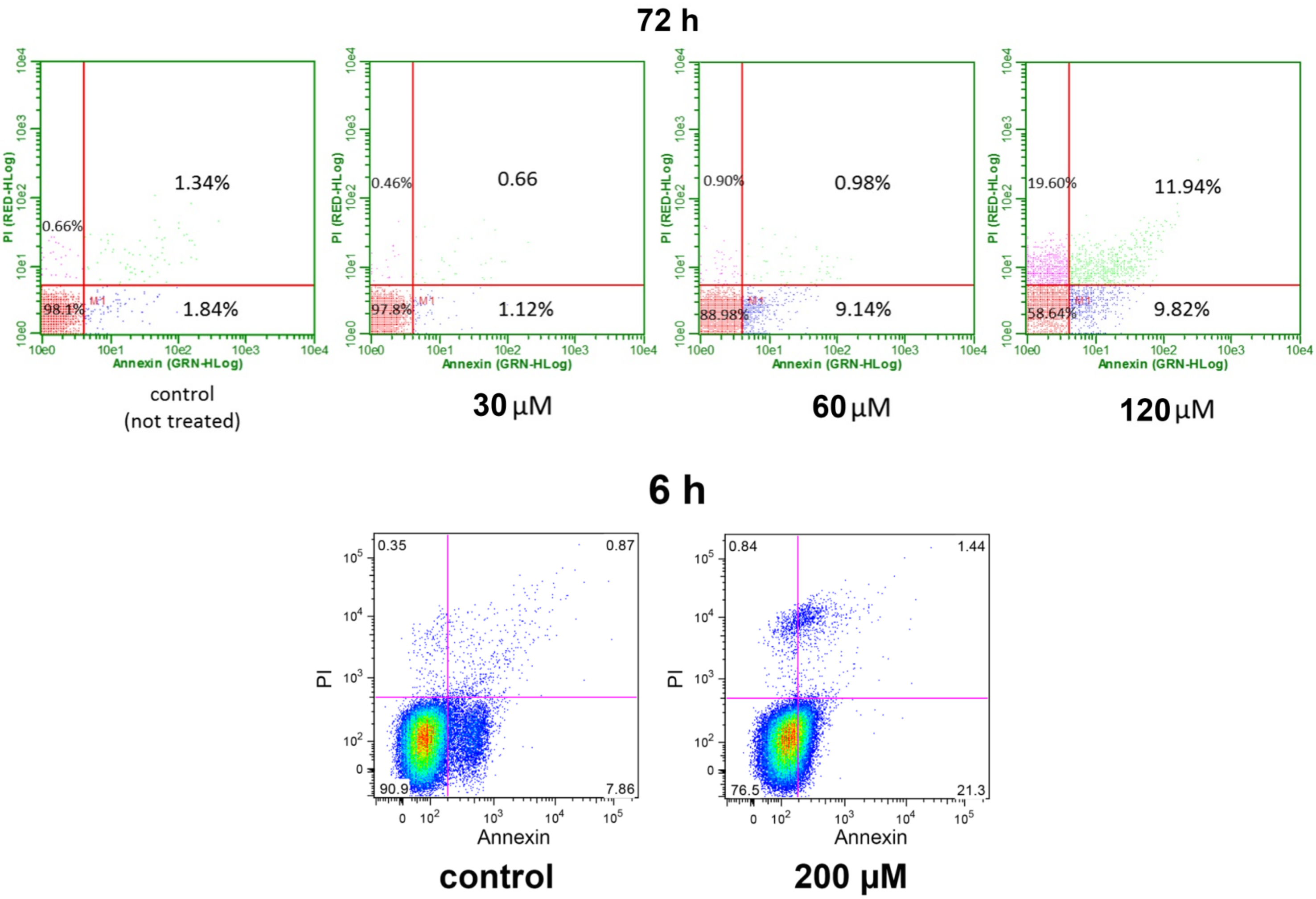

S3 Fig. Apoptosis and membrane stability analysis. Dot-plot graphics of flow cytometry analysis of annexin-V labeling (x-axis) and propidium iodide (y-axis) were performed in order to evaluate apoptosis and the plasma membrane permeability of parasites treated with 30, 60 and 120 µM of isotretinoin for 72 h (upper panel), 200 µM of drug for 6 h (lower panel) and untreated cells (control). The results suggest that the plasma membrane remains unaltered after isotretinoin exposure and necrotic processes were not observed in any treatment. Only the parasites exposed to 200 µM for 6 h were positive for annexin V.
