# Supplementary material for: Trypanocidal Effect of Isotretinoin through the Inhibition of Polyamine and Amino Acid Transporters in Trypanosoma cruzi
Source: PLoS Negl Trop Dis. 2017 Mar 17;11(3):e0005472. doi: 10.1371/journal.pntd.0005472 (PMC5371382; doi:10.1371/journal.pntd.0005472)
Supplement: S4 Fig — Extraction experiments were carried out using isotretinoin (A) or the non-ionic detergent digitonin as extraction control (B). 108 parasites were incubated with increasing amounts of isotretinoin (5, 25 and 100 μM), or digitonin (0 and 0.3 mg.mL-1). Fractionation results were analyzed by Western blot using antibodies anti- T. cruzi glutamate dehydrogenase which localizes in the parasites cytosol. Upper and lower lines of each figure showed the pellet and supernatant fractions after extraction, respectively. Numbers above each figure indicate the concentration of isotretinoin (A) or digitonin (B). The detailed protocol was described under Methods. (PDF) [file pntd.0005472.s004.pdf]

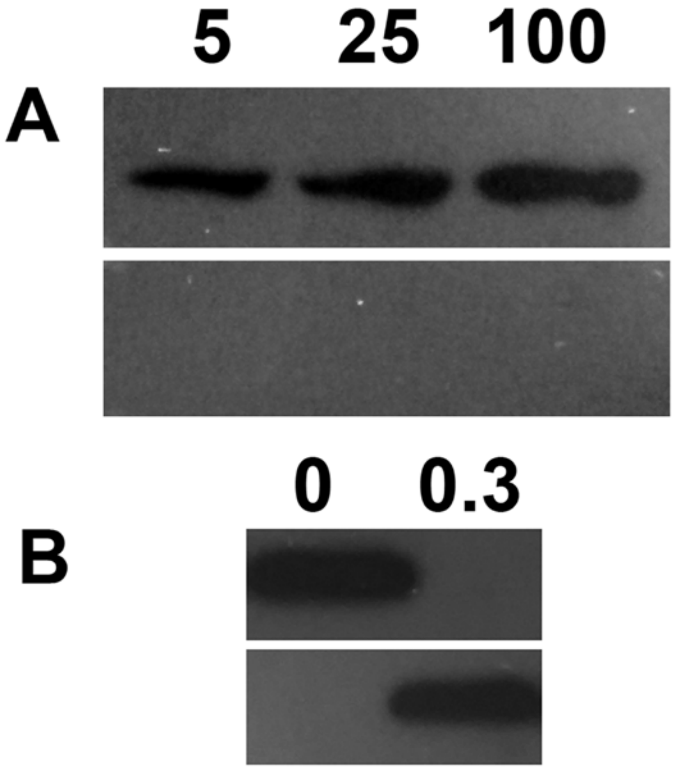


**S4 Fig. Effect of isotretinoin on the plasma membrane.** Extraction experiments were carried out using isotretinoin (A) or the non-ionic detergent digitonin as extraction control (B). $10^8$ parasites were incubated with increasing amounts of isotretinoin (5, 25 and 100 μM), or digitonin (0 and 0.3 $mg.mL^{-1}$). Fractionation results were analyzed by Western blot using antibodies anti- *T. cruzi* glutamate dehydrogenase which localizes in the parasites cytosol. Upper and lower lines of each figure showed the pellet and supernatant fractions after extraction, respectively. Numbers above each figure indicate the concentration of isotretinoin (A) or digitonin (B). The detailed protocol was described under Methods.
